# Lateral elbow magnetic resonance imaging findings in patients without pain complaints

**DOI:** 10.1016/j.jpra.2024.06.005

**Published:** 2024-06-12

**Authors:** Masaomi Saeki, Hidemasa Yoneda, Michiro Yamamoto

**Affiliations:** Department of Human Enhancement & Hand Surgery, Nagoya University Graduate School of Medicine, Nagoya, Japan

**Keywords:** Magnetic resonance imaging, Lateral elbow, Lesion, No elbow pain, Lateral epicondylitis

## Abstract

Magnetic resonance imaging (MRI) can help evaluate lateral epicondylitis; however, abnormal findings on MRI are not always consistent with the symptoms. The occurrence of such abnormal MRI findings at the lateral side of the elbow in patients without pain remains unclear. Therefore, the purpose of this study was to investigate the MRI findings of the lateral elbow joint in patients with no complaints of pain in the elbow joint.

We retrospectively identified 152 patients who had undergone MRI of the area including the elbow from July 2015 to January 2022. We excluded patients with pain in the elbow area and those with diagnosis of diseases that could affect MRI findings at the lateral elbow. The presence of lateral collateral ligament complex (LCLC) and common extensor tendon (CET) lesions on MRI was assessed by two reviewers.

In total, 22 patients (12 men and 10 women) were included in the analysis. The mean age of the patients was 54 years. Five patients, all ≥65 years old, had abnormal findings related to the LCLC or CET on MRI. Abnormal LCLC and CET findings on MRI can be encountered in older patients even in the absence of elbow pain.

## Introduction

Lateral epicondylitis is a common musculotendinous disorder of the extensor muscles of the forearm and the lateral epicondyle of the humerus, usually affecting middle-aged patients.^1^, ^2^, ^3^ Its typical symptoms include pain at the lateral side of the elbow, and its diagnosis is based on these symptoms and physical examination including the Thomsen and middle finger extension tests.^4^^,^^5^ Magnetic resonance imaging (MRI) often aids in evaluating the intraarticular state, other causes of pain, and extent of the lesion for surgical treatment.^6^^,^^7^ However, MRI findings of the elbow are not always consistent with the clinical evaluation of the patient. In fact, a clinical study on patients with lateral epicondylitis of the elbow showed that the changes identified on MRI were persistent at 6 weeks of follow-up despite overall clinical improvement.^8^

We aimed to address whether abnormal findings on MRI are related to lateral epicondylitis in a clinically diagnosed patient, and more appropriately to clarify the occurrence of abnormal MRI findings in patients without any symptoms pertaining to the elbow. Two clinical studies have investigated the healthy contralateral side of patients or healthy volunteers to report on the MRI findings of the lateral elbow in subjects without complaints of pain at the elbow.^8^^,^^9^ However, the subjects in those studies were either younger than those in whom lateral epicondylitis commonly occurs or the description of the physical findings of the contralateral side was poor. Owing to the limitations of the previous studies, the MRI findings of the lateral side of the elbow in patients without pain complaints are yet to be clearly elucidated.

The purpose of this study was to investigate the MRI findings of the lateral elbow joint in patients who had no complaints of pain in the elbow joint.

## Patients and methods

### Ethical statements

The study was conducted in compliance with the Declaration of Helsinki, approved by the Nagoya University Graduate School of Medicine's ethics authority, and conducted in accordance with the policies and regulations of our institution. We used previously collected patient data; therefore, information disclosure documents were released without the need for individual consent.

### Patient selection and participant characteristics

We used medical records and imaging data from two different hospitals. We retrospectively studied patients who underwent MRI scans of the area of interest including the elbow from July 2015 to January 2022. In total, 152 patients were enrolled. The decision to perform an MRI examination was made by an orthopedic surgeon.

In this study, we excluded patients whose diagnosed disease before or after the MRI scan could affect the MRI findings at the lateral elbow, cases of inadequate documentation of clinical findings in the medical record, patients who experienced pain in the elbow area, and cases wherein the image sections of the elbow area did not facilitate evaluation of the MRI findings.

The data obtained from the medical records included the patients’ age at the time of the MRI scan, sex, diagnosis after the MRI scan, and symptoms of elbow pain.

### Image assessment and measurements

We assessed the presence of lateral collateral ligament complex (LCLC) and common extensor tendon (CET) lesions on MRI and used the Baker classification. MRI was performed in all patients using a 1.5T or 3.0T system (MAGNETOM Aera 1.5T and MAGNETOM Avanto 3.0T, Siemens Healthineers, Erlangen, Germany). The LCLC and CET were evaluated on T2-weighted or short tau inversion recovery (STIR) axial and coronal images.

Based on the MRI findings of coronal images, LCLC lesions were classified as follows: LCLC0, normal status; LCLC1, partial tear, thickening or thinning of the ligament; and LCLC2, near-complete or complete tear. Similarly, CET lesions were classified as follows: CET1, tendinopathy or low-grade partial tears; CET2, intermediate-grade partial tears; and CET3, high-grade partial or complete tears.

In accordance with the criteria used in previous studies, a low-grade partial tear was defined as an injury with <20% tendon thickness, an intermediate-grade partial tear represented an injury with 20%–80% tendon thickness, and a high-grade partial tear reflected an injury with >80% tendon thickness. A full tear was defined as an injury without continuity and with the attachment of the lateral epicondyle of the humerus.^6^^,^^10^^,^^11^

### MRI finding reviews

We reviewed the MRI findings from September 2022 to February 2023. Two authors independently reviewed the findings and determined the disease grade following each MRI finding. Discrepancies in the evaluation were resolved through discussion. Inter-reviewer agreement on the grading of MRI findings was evaluated using the kappa coefficient and categorized as follows: near-perfect (0.81–1.00), substandard (0.61–0.80), moderate (0.41–0.60), fair (0.21–0.40), and poor (0.00–0.20). The authors are board-certified by the Japanese Society for Surgery of the Hand.

### Statistical analysis

We calculated the intraclass correlation coefficients for the Baker classification and MRI findings of LCLC and CET. All statistical analyses were performed using IBM SPSS Statistics, version 28 (IBM Corporation, Armonk, New York, USA).

## Results

### Selection

**Figure 1:**
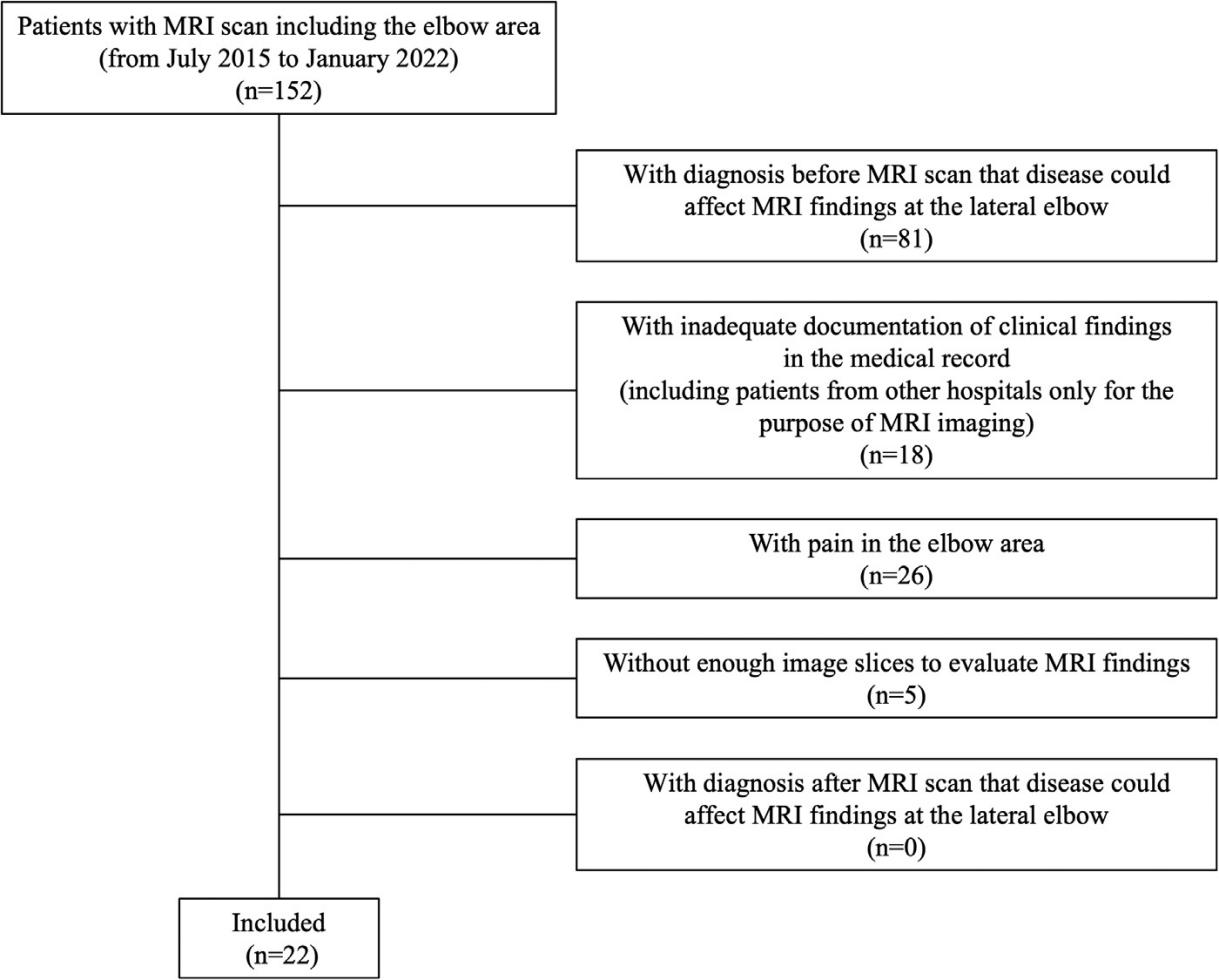
Selection process for eligible patients. MRI, magnetic resonance imaging.

Figure 1 presents the patient selection process according to the aforementioned criteria. Patients with diagnoses, such as lateral epicondylitis, medial epicondyle apophysitis, crush injury of the elbow, traumatic hematoma of the elbow joint, elbow contusion, lateral or medial collateral ligament injury of the elbow joint, dog bite injury, arthritis or septic arthritis of the elbow, cellulitis of the elbow or forearm, synovia osteochondromatosis of the elbow, elbow dislocation, humerus transcondylar fractures, proximal humeral fracture, traumatic hematoma, radial head fracture, and radial neck fracture, before the MRI scan were excluded as the MRI findings at the lateral elbow may have been affected:. The MRI findings of 22 patients were included in the investigation (Fig. 1).

### Patients

Table 1 presents the patients’ baseline characteristics. The mean age was 54 years (range, 13–89 years), with 12 men and 10 women patients. The diagnoses after undergoing MRI were as follows: 15 patients had a mass at the site of or around the elbow (6 in the medial, 3 in the anterior, 5 in the posterior, and 1 in the distal humerus), 3 patients had a cubital tunnel syndrome, and 1 patient with each of the following was identified: carpal tunnel syndrome, median nerve injury, radial neuropathy, and cervical spondylosis (Table 1).

**Table 1:** Characteristics of patients with no complaints of pain at the elbow joint included in the investigation of MRI findings.

Variable	
Age (years)	54 ± 21
Sex (male, %)	12 (55)
Diagnosis after MRI scan	
Tumor	15
Location (cutaneous/subcutaneous)	
Medial elbow	6 (1/5)
Anterior elbow	3 (2/1)
Posterior elbow	5 (1/4)
Distal humerus	1 (1/0)
Cubital tunnel syndrome	3
Carpal tunnel syndrome	1
Median nerve injury	1
Radial neuropathy	1
Cervical spondylosis	1
Cases with abnormal MRI finding	5 (22)

Data are presented as n (%) or mean ± standard deviation.

MRI, magnetic resonance imaging.

### Patients with abnormal MRI findings

All the included patients underwent MRI using a 1.5T system. Patients who underwent an MRI using a 3.0T system were excluded during the selection process. Imaging was performed in the axial, coronal, and sagittal planes for 16 patients; in the coronal and sagittal planes for 4 patients; and in the coronal and axial planes for 2 patients.

The parameters for imaging were as follows: the repetition time (TR) and echo time (TE) of the T1-weighted sequence were 440–570 and 12–14 ms, respectively. The TR and TE of T2-weighted sequence were 3500–4010 and 63–100 ms, respectively. The TR, TE, and T1 of the STIR sequence were 3400–7690, 59–76, and 170 ms, respectively. The TR and TE of T2-weighted fat suppression sequence were 3500–4460 and 69–73 ms, respectively. The TR and TE of T2-weighted signal targeting with alternating radiofrequency sequence were 400–781 and 19 ms, respectively. The field of volume was 120–150 mm for the axial, 97–200 mm for the coronal, and 82–150 mm for the sagittal planes. The thickness of the axial, coronal, and sagittal planes was 3–5, 3–4, and 3–4 mm, respectively.

**Figure 2:**
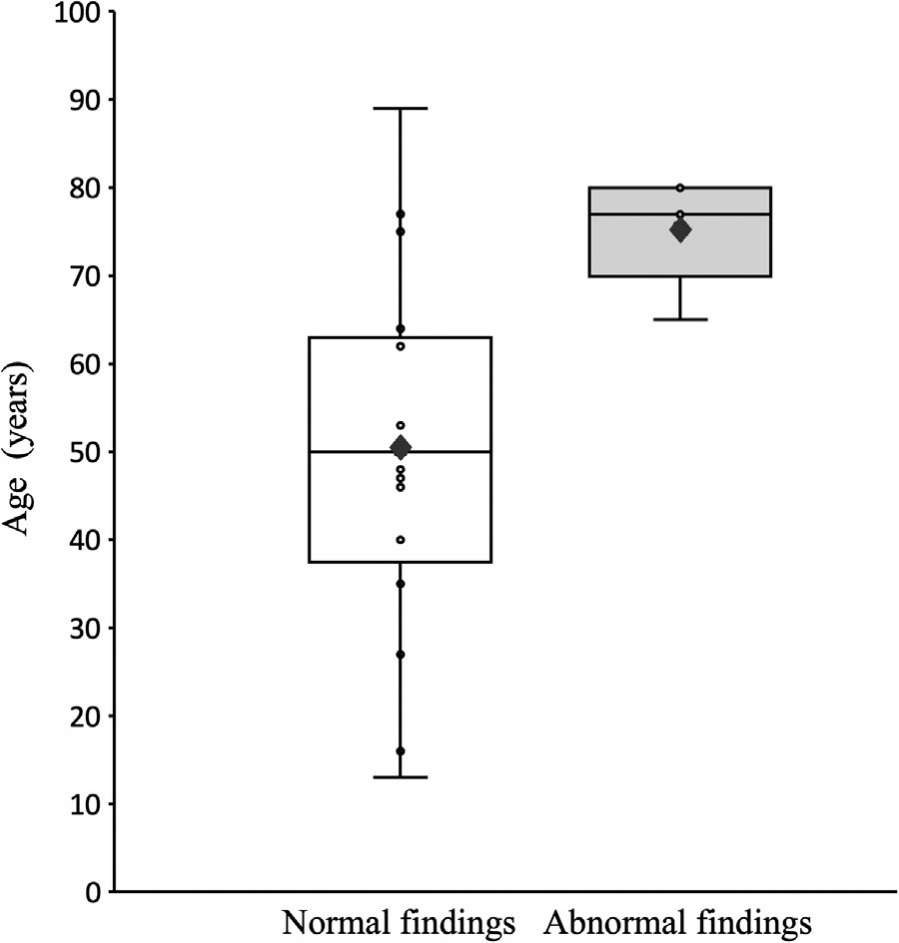
Ages of patients with normal and abnormal MRI findings. In this box plot of the patients’ age with normal and abnormal MRI findings, diamonds indicate the means; horizontal bars in the box indicate the medians; the tops and bottoms of the boxes indicate the upper and lower quartiles, respectively; the whiskers indicate ±1.5 times the interquartile range or the smallest or highest value; and the dots indicate individual values. MRI, magnetic resonance imaging.

Abnormal MRI findings were detected in 5 patients. The mean age of the patients with abnormal MRI findings was 75 years, and all of them were older adults (≥65 years old [range, 65–80 years]) (Fig. 2); 2 patients were men and 3 were women. The diagnoses after MRI were as follows: a mass at the site of or around the elbow in 4 patients and carpal tunnel syndrome in 1 patient. Absence of elbow pain was clearly stated in the medical record of 4 patients, and for 1 patient, the palpation of a mass was the only symptom mentioned.

The LCLC lesions were classified as LCLC0, LCLC1, and LCLC2 in 2, 1, and 1 patient, respectively; the CET lesions were classified as CET1, CET2, and CET3 in 2, 1, and 1 patient, respectively. For 1 patient, the MRI coronal slices were not available; therefore, no classification was performed (Table 2).

**Table 2:** Characteristics of patients with abnormal MRI findings and their classification.

Case	Age (years)	Sex	Diagnosis after MRI	No elbow pain	LCLC	CET
1	80	Female	Carpal tunnel syndrome	+	LCLC2	CET3
2	75	Female	Tumor (medial)	+	LCLC0	CET1
3	77	Female	Tumor (medial)	-a	-b	-b
4	80	Male	Tumor (posterior)	+	LCLC1	CET1
5	65	Male	Tumor (medial)	+	LCLC0	CET2

Classification of LCLC lesions: LCLC0, normal status; LCLC1, partial tear, thickening or thinning of the ligament; LCLC2, near-complete or complete tear. Classification of CET lesions: CET1, tendinopathy, or low-grade partial tear; CET2, intermediate-grade partial tear; CET3, high-grade partial or complete tear.

MRI, magnetic resonance imaging.

a Case 3 was described only as palpation of a mass and no other symptoms.

b No evaluation for the classification of LCLC and CET was performed in case 3, because the patient bore no coronal MRI section. LCLC, lateral collateral ligament complex; CET, common extensor tendon.

### Validity of the MRI classification

All inter-reviewer MRI classifications exhibited substantial agreement, with a kappa value of 0.67.

## Discussion

We demonstrated that, among the 22 patients included in our retrospective study, 5 (≥65 years old) showed abnormal MRI findings of CET and LCLC without complaints of elbow pain. This indicates that CET and LCLC abnormalities on MRI are not always accompanied by the symptom of pain. In patients with lateral epicondylitis, abnormal findings on MRI could be observed before the onset of pain, particularly in older patients. Our findings suggest that diagnosis and treatment should be considered based on the clinical findings and not on imaging results, especially in older patients. Information on the presence of asymptomatic abnormal findings on MRI could help clinicians to cautiously plan surgery because MRI findings are primarily used for surgical planning and rarely for diagnosis.

In previous studies, MRI findings of the elbows in the absence of disease or trauma have been reported only as a comparison group for diseases such as lateral epicondylitis.^9^^,^^12^, ^13^, ^14^ In those studies, the elbows of volunteers or contralateral elbows of patients with epicondylitis were included. However, the weighted mean age of the included volunteers was 29 years, resulting in poor comparability in terms of age, considering that the peak prevalence age of lateral epicondylitis is 45 years, with most patients being 35–54 years old.^15^^,^^16^ Moreover, the documentation of the patients’ clinical records for the contralateral elbows was poor. In this study, we excluded patients with inadequate documentation of clinical findings in the medical record, and patients of various ages (average age, 54 years) were included.

In a previous study, elbow MRI was performed in patients with extensor carpi radialis brevis (ECRB), lateral collateral ligament abnormalities, and in matched controls; however, the control patients were not asymptomatic.^17^ In our study, patients with pain in other areas or near the elbow, not limited to the lateral side of the elbow, were excluded to ensure the absence of pain at the lateral elbow. To our knowledge, this is the first cross-sectional study to investigate the MRI findings of CET and LCLC in patients without elbow pain.

In the present study, abnormal findings of CET or LCLC on MRI were found only in patients aged ≥65 years (5 out of the 8 patients who were 65 years or older). However, the cause of this high incidence of asymptomatic lesions of CET or LCLC in older patients is not clear. For rotator cuff tendinosis of the shoulder, asymptomatic MRI abnormalities were seen commonly in older patients and considered to be related to degenerative changes.^18^^,^^19^ A clinical study using MRI for preoperative evaluation of ECRB suggested that degenerative changes were associated with asymptomatic enthesopathy.^17^ Our study indicated that asymptomatic abnormal findings of CET or LCLC on MRI could be related to degeneration caused by aging.

The MRI of all patients included in this study was not performed using 3.0T but by using 1.5T, as high magnetic field intensity MRI is expected to improve the signal-to-noise ratio and image resolution. Nevertheless, clinical studies on knee MRI have shown no significant difference in the accuracy of knee menisci and anterior cruciate ligament evaluation between the 1.5T and 3.0T systems.^20^^,^^21^

Our study had a few limitations. First, this study had a retrospective design, while a prospective cohort study could have provided a higher level of evidence. Second, the data did not contain any information regarding the pain and MRI results for 1 patient with abnormal MRI findings due to the absence of a coronal MRI section. Only a positive description of a mass palpation and no other symptoms were present in the patient's medical record. Nevertheless, this finding was not expected to significantly affect the results of this study because the mention of “no other symptoms” is considered to imply the absence of pain. Third, the position of the arm was not uniformly defined when the patients were examined. Fourth, the number of patients included is insufficient to generalize the applicability of the observations. The data in this study were obtained from 22 carefully selected patients after implementing strict exclusion criteria on a starting sample of 152 patients. Despite these limitations, we believe that the findings of this study constitute an important dataset for the evaluation of MRI findings in lateral epicondylitis.

## Conclusions

Our analysis suggests that LCLC and CET abnormal findings on MRI could be found in older patients even in the absence of elbow pain.

## Conflict of interest

None.
